# Influence of Enteral Nutrition on Quality of Life in Head and Neck Cancer and Upper Gastrointestinal Tract Cancer Patients within a Pair-Matched Sample

**DOI:** 10.3390/nu15214698

**Published:** 2023-11-06

**Authors:** Elwira Gliwska, Dominika Głąbska, Zuzanna Zaczek, Jacek Sobocki, Dominika Guzek

**Affiliations:** 1 Department of Food Market and Consumer Research, Institute of Human Nutrition Sciences, Warsaw University of Life Sciences (WULS-SGGW), 159C Nowoursynowska Street, 02-776 Warsaw, Poland; dominika_guzek@sggw.edu.pl; 2 Cancer Epidemiology and Primary Prevention Department, Maria Sklodowska-Curie National Research Institute of Oncology, 15B Wawelska Street, 02-034 Warsaw, Poland; 3 Department of Dietetics, Institute of Human Nutrition Sciences, Warsaw University of Life Sciences (WULS-SGGW), 159C Nowoursynowska Street, 02-776 Warsaw, Poland; dominika_glabska@sggw.edu.pl; 4 Department of General Surgery and Clinical Nutrition, Centre of Postgraduate Medical Education in Warsaw, 231 Czerniakowska Street, 00-416 Warsaw, Poland; zuzanna.zaczek@wum.edu.pl (Z.Z.); jsobocki@mp.pl (J.S.); 5 Department of Human Nutrition, Faculty of Health Sciences, Medical University of Warsaw, 27 Erazma Ciolka Street, 01-445 Warsaw, Poland

**Keywords:** quality of life, cancer, enteral nutrition, EORTC QLQ C30

## Abstract

Patients with cancers of the head and neck and upper gastrointestinal tract are particularly susceptible to malnutrition, which worsens both their prognosis and quality of life and may result in the need for enteral or parenteral nutrition. The aim of this study was to investigate the impact of enteral nutrition on the quality of life in a paired sample. This study included 50 patients with cancer in two paired subgroups: with enteral nutrition (studied group) and without enteral nutrition (matched group). This study was based on self-reports collected with the EORTC QLQ C30 questionnaire and retrospective analysis of medical records. The analysis revealed that weight loss, group type, and age were the primary factors influencing patients’ quality of life. Compared with all cancer patients and the general Polish population, the scores of patients in both groups were below reference values for functional scales and exceeded reference values or were similar for fatigue and vomiting/nausea. Patients who received enteral nutrition more frequently scored lower on the functional scales and higher on the symptomatic scales than the control group. These findings emphasize the complex relationship between cancer, nutritional status, and quality of life.

## 1. Introduction

According to the Global Cancer Statistics (GLOBOCAN) for the year 2020, it was re-ported that there were approximately 19.3 million new cancer cases and nearly 10.0 million cancer-related deaths [1]. It is predicted that the number of new cancer cases will continue to increase, which shows that cancer is a major public health problem [2]. Numerous factors related to cancer and its treatment can negatively affect patients’ quality of life [3].

Cancer patients face various problems, such as gastrointestinal symptoms, hair loss, taste and smell disorders, pain, and unintentional weight loss. The patient’s quality of life may also be affected by weakness, loss of muscle mass and strength, fever, or other com-plications specific to the cancer site. In addition, cancer patients are compelled to undergo demanding medical protocols and stay in hospital wards or outpatient facilities. Staying in the hospital means a break from their usual routine, isolation from social contacts, and sometimes separation from their professional obligations. These conditions can create feelings of loneliness and anxiety and separate patients from their relatives. For cancer patients in particular, these feelings can be exaggerated and have a negative impact on quality of life [4]. Recognizing the importance of patients’ quality of life, there is increasing emphasis on addressing these issues alongside treatment itself [5]. Efforts are being made to prevent and alleviate factors that worsen quality of life [6]. Particular attention is being paid to dietary care and appropriate clinical nutrition in cancer treatment facilities. Vomiting, nausea, weight loss, and malnutrition are the most important nutrition-related complications in cancer patients.

Currently, it is recommended that the nutritional status of patients be reviewed regularly from diagnosis to completion of treatment [7]. It is recommended to diagnose malnutrition as well as its severity; given the specific pathologic mechanisms of cancer, cancer patients are at high risk of developing malnutrition, which can significantly affect their prognosis [8]. Malnutrition affects the vast majority of cancer patients, with estimates ranging from 20% to over 70% [9]. Surprisingly, malnutrition itself accounts for approximately 20% of cancer-related deaths, underscoring its significant impact [10]. Cancer-related malnutrition is a complex phenomenon, as numerous elements come together to impede food intake, increase dietary requirements and nutrient loss, and decrease anabolic triggers such as physical activity, as well as alter metabolic functions in various organs or tissues. The various factors contributing to malnutrition form the basis for a number of treatment approaches aimed at addressing malnutrition in cancer patients [11,12]. In many cases, it is not possible to meet an individual’s nutrient needs through regular diet alone. Therefore, there is a clear need for specialized nutritional care in oncology. Nutritional counselling and the use of oral nutritional supplements can help many cancer patients [13]. However, when meeting nutritional needs through normal diet is not possible or not sufficient, enteral or parenteral nutrition is required. In addition, commonly occurring dysphagia, or treatment complications in the head and neck region are among the indications for administration of clinical nutrition [14,15]. It is important to note that both enteral and parenteral nutrition can be safely administered at home after appropriate training in specialized settings. Extensive research has shown that nutritional support, including dietary counselling, oral nutritional supplements, and enteral or parenteral nutrition, positively affects the efficacy of cancer treatment, overall survival, and the quality of life of patients [14]. However, it is important to note that patient-centred care is required, taking into account individual needs, abilities, communication, and various aspects of quality of life [16]. Enteral nutrition might cause complications that would negatively influence quality of life, and the most common are complications related to the gastrointestinal tract, including constipation, diarrhoea, regurgitation, or abdominal distension [17]. Additionally, many complications related to emotional status and social life have also been identified in the previous works. Patients with cancer and enteral feeding may experience, for example, distancing of relationships, social stigma, sense of non-acceptance, or internal conflicts [18]. Considering the significant role that enteral nutrition plays in cancer treatment, it is important to evaluate its impact on the quality of life of cancer patients. With this in mind, the aim of this study was to evaluate the impact of enteral nutrition on the quality of life of patients with head and neck and upper gastrointestinal tract cancers in a paired sample.

## 2. Materials and Methods

### 2.1. Ethical Statement

This study was conducted based on the guidelines of the Declaration of Helsinki. Each patient was informed of the purpose and nature of the research and gave informed consent. Bioethics Committee approval to conduct the study was obtained from the Bio-ethics Committee of the Medical Centre for Postgraduate Education in Warsaw, Poland, on 14 July 2021, Order 116/2018.

### 2.2. Study Design and Population

This study included a group of 50 patients diagnosed with cancer of the head and neck or upper gastrointestinal tract in 2 paired subgroups—with enteral nutrition (studied group) and without enteral nutrition (matched group). The detailed description of the study group has been presented in Table 1. Patients were enrolled in the study during their hospitalization at the Gastroenterology Unit in the Oncological Hospital (Warsaw, Poland) or during their quarterly visits to the Polish Outpatient Clinic for Parenteral and Enteral Nutrition (Warsaw, Poland) between January 2022 and September 2023. Patients were recruited to the study through purposive sampling. The inclusion criteria for the study were as follows: aged over 18 years, confirmed cancer diagnosis, enteral nutrition with active enteral access (in the studied group), Polish as native language, linguistically and cognitively competent, and consent to participate in the study. Exclusion criteria were as follows: missing information in the questionnaire, nonsensical information in the questionnaire, parenteral nutrition, refusal of nutritional care, unclear diagnosis, enteral nutrition providing less than 50% of estimated energy needs (in the studied group), and previous enteral or parenteral nutrition (matched group). Interviews with patients were conducted by an interviewer who was a dietitian experienced in the field studied. The pairing criteria were as follows: cancer site, gender and age ±5 years.

In the study group, all patients received specific enteral nutrition formulas. The energy value and macronutrients content of the nutritional formulas were determined individually for each patient by the nutrition team, but the main determinant of the amount of nutrient mixture administered was the clinical condition of the patient.

### 2.3. Interviews

The study was based on self-reports collected using the EORTC QLQ C30 questionnaire, while all other information about the cancer, its course and treatment, and body weight and height were obtained from medical records.

The EORTC QLQ C30 questionnaire is a validated questionnaire for assessing health-related quality of life in cancer patients with physical, psychological, and social functioning and was used in its validated Polish version [19]. It consists of nine multi-item scales: five functioning scales (physical, role, cognitive, emotional, and social), a global quality of life scale, and three symptom scales (fatigue, pain, and nausea/vomiting). In addition, six individual symptom scales are used (dyspnea, insomnia, loss of appetite, constipation, diarrhoea, and financial difficulties). Version 3.0 is the standard version of the EORTC QLQ C30, with four-item scales for the 28 items used and seven-item scales for assessing general health and quality of life in the past week. The items on the four-item scale take values from 1 to 4, giving a range of 3, while the items on the seven-item scale take values from 1 to 7, giving a range of 6. All scales and individual items are linearly transformed into a 0–100 scale.

In addition, self-reported socioeconomic data were collected along with the EORTC QLQ C30 questionnaire. The collected socioeconomic data were as follows:

- Gender;
- Age;
- Educational level (higher education, secondary education, vocational education, or primary education);
- Place of residence (rural area, city with up to 20,000 inhabitants, city with 100,000 to 500,000 inhabitants, city with 20,000 to 100,000 inhabitants, or city with more than 500,000 inhabitants);
- Employment (full-time employment, part-time employment, temporary employment, pension, retirement, or unemployment);
- Economic situation (very good, good, neither good nor bad, bad, very bad, or difficult to say).

Weight loss was calculated based on current weight at clinic visits and self-reported weight loss in the last six months. Body mass index (BMI) was calculated by dividing weight (kg) by height (m) squared. The overall nutritional status was also assessed for each patient based on unintentional weight loss in the past six months, decreased food intake, and ongoing course of the disease. The nutritional status was assessed by a qualified clinical dietitian based on responses given in the questionnaire and patient documentation analysis.

To evaluate the results of EORTC QLQ C30, reference values for the functioning scale and global health status/quality of life were taken from the EORTC Quality of Life Group Members’ Manual and other users of the QLQ C30 [20]—reference scores for all cancer patients at all stages and for the general population normative data for the EORTC QLQ C30 Health-Related Quality of Life Questionnaire based on 15,386 individuals from 13 European countries [21]. The review by Grulke et al. [22] pointed out that regardless of the proposed rules of thumb for interpreting differences between two scores on the QLQ C30 (of 5–10, 10–15, or >20 points), the clinical setting depends on the clinical context, and therefore, no general rule can be given.

### 2.4. Statistical Analysis

Normality of the distribution was tested using the Shapiro–Wilk test. Comparisons between groups were made using chi^2^ test, Student’s *t*-test (for parametric distributions) and the Mann–Whitney U-test (for nonparametric distributions). Pearson correlation coefficient (for parametric distributions) and Spearman rank correlation coefficient (for nonparametric distributions) were used to analyse the correlations between the EORTC QLQ C30 scales. The reliability (internal consistency) of the EORTC QLQ C30 scales was assessed using the Cronbach’s alpha coefficient. A value of ≥0.70 was considered appropriate [23].

In addition, the stepwise multiple regression analysis was performed in a model that included the type of group (studied or matched group), gender, age, education level, place of residence, employment, economic situation, and weight loss, and the unstandardized and standardized β-coefficients and *p*-values were presented. The level *p* ≤ 0.05 was treated as statistically significant. Statistical analysis was performed using Statistica 13.3 (StatSoft Inc., Tulsa, OK, USA).

## 3. Results

The sociodemographic characteristics of the patients are shown in Table 1. The majority of the patients studied had an educational level no higher than secondary school, were retired, lived in rural areas or small towns, and reported having at least a good economic status. There were no differences between the studied and matched groups in age, gender, education level, residence, and employment status, while a statically significant difference was found only for economic status (*p* = 0.0323), as the higher proportion of the studied group classified their status as neither good nor bad, while in the matched group, the higher proportion explicitly chose good/very good or bad/very bad. Most patients were diagnosed with cancers of the larynx (14%) and oesophagus (10%), but cancers of the nasopharynx, stomach, and others were also diagnosed among patients enrolled in the study. According to the ICD-10 classification, the following cancer sites were identified: C04, C09, C11, C15, C16, and C32 [24]. Most of the participants in the study were diagnosed with malnutrition. The average BMI was 21.5 kg/m² with a standard deviation (SD) of 4.499. Additionally, the average percentage of weight loss over the past 6 months was 10.69% (SD 9.368), and the average kilogram weight loss during the same period was 10 kg (SD 5.875). The mean age of patients was 63 years (11.8 SD), with no differences between groups (Table 2).

**Figure 1:**
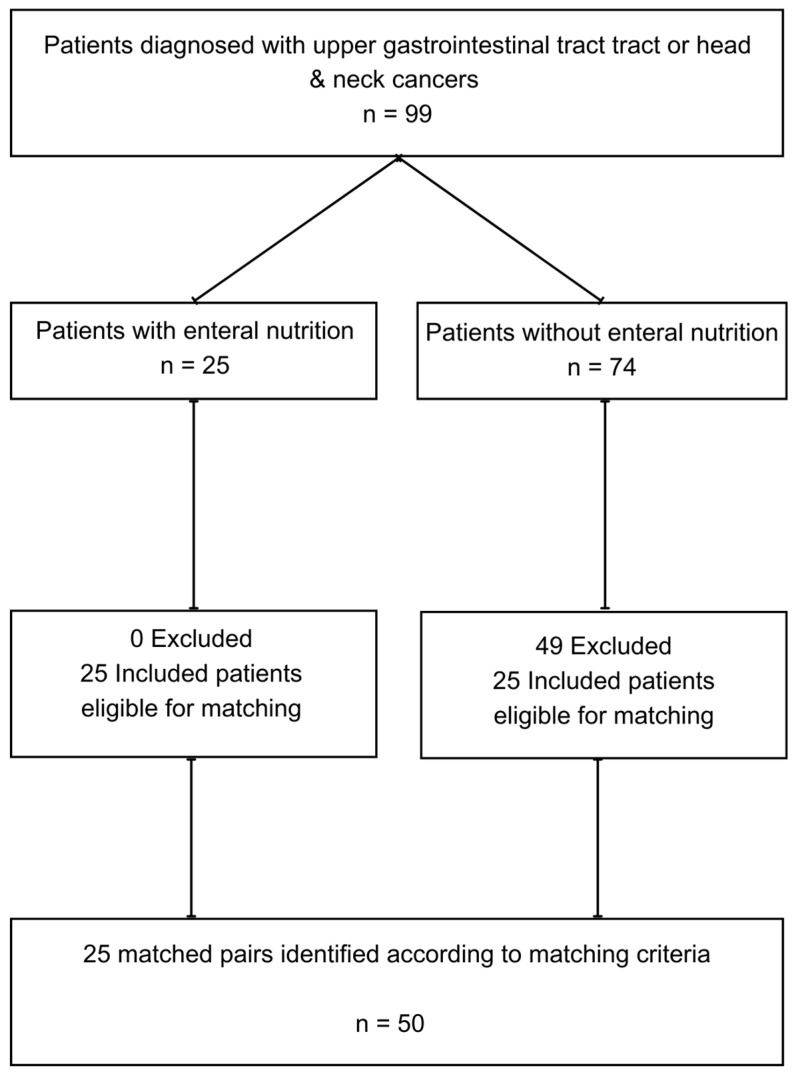
The process of selecting matched pairs for the study.

**Figure 2:**
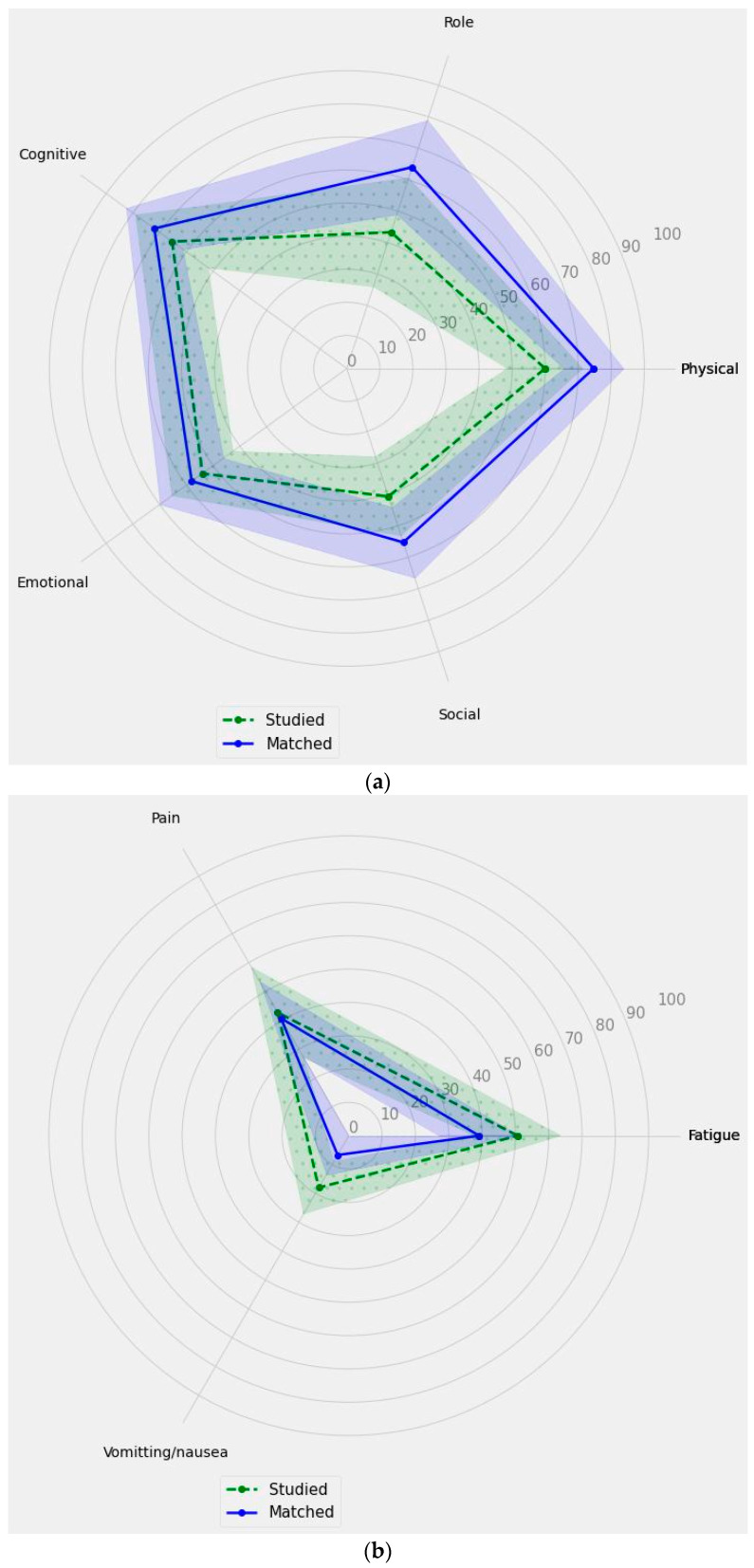

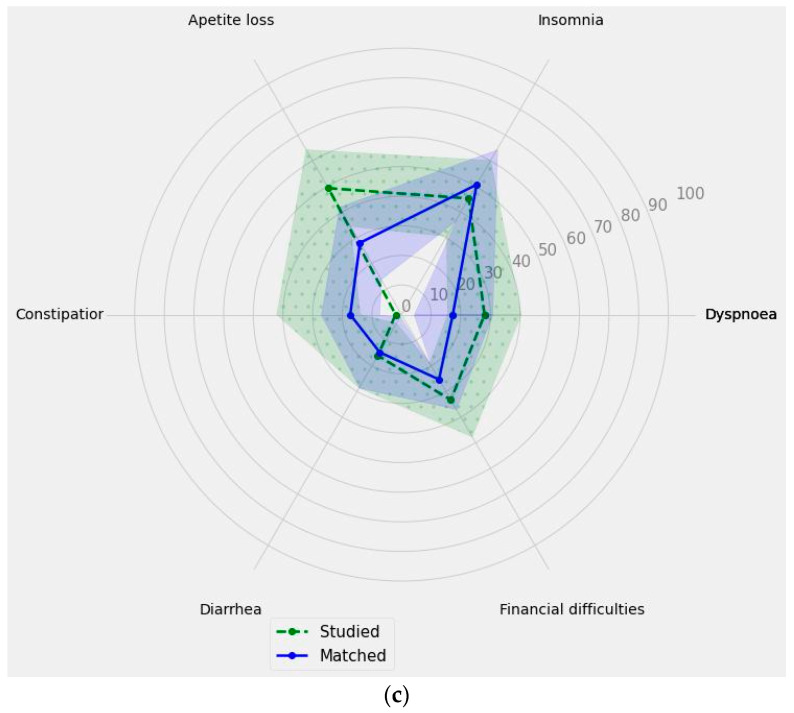
The comparison of the main subscales’ measurements of the EORTC QLQ C30 for the studied group and matched group. The solid line represents the results obtained in points for individual subscales within the matched group, while the dashed line corresponds to the results obtained in points for individual subscales within the study group. The shaded areas indicate the confidence intervals (CIs): (**a**) functional scales; (**b**) symptom scale; (**c**) single-items measures score.

Table 3 shows the descriptive statistics and scale reliability of the EORTC QLQ C30, while Table 4 shows the item-scale correlation matrix. For 6 scales (for both groups), the mean or median values (depending on the distribution) were below the reference values for both cancer patients [20] and the general Polish population [21], except for fatigue, vomiting/nausea, dyspnoea, insomnia, constipation, and diarrhoea, for which the obtained scores were similar to (or better than) the reference values, and for pain, loss of appetite, and financial difficulties, for which the obtained scores were above the reference values (better results). Figure 1 shows the comparison of the functional scales for the studied group and the matched group. It can be seen that the scores of the physical scale for the studied group were significantly lower (worse) than the scores of the matched group (*p* = 0.0353). Figure 2 shows the comparison of symptom scales for the studied group and matched group. It can be seen that the values in the vomiting/nausea (*p* = 0.0446) and loss of appetite (*p* = 0.0416) scales for the studied group were significantly higher (better) than the values for the matched group.

Figure 2 shows the comparison of the three main subscales’ measurements of the EORTC QLQ C30 for the studied group and matched group, and it showed that patients from the studied group differ from the matched group in several subscales of the questionnaire.

As shown in Figure 2a, the studied group exhibited lower scores in all functional subscales when compared to the matched group. Furthermore, Figure 2b demonstrates higher scores in symptomatic scales for the studied group, which correlated with more frequent experiences of certain symptoms—fatigue, vomiting or nausea, and pain. Moreover, Figure 2c depicts variations in scores between the studied group and the matched group, with some scales showing higher scores for the studied group (constipation and insomnia) and others showing lower scores (financial difficulties and appetite loss).

The results of the stepwise multiple regression analysis for the EORTC QLQ C30 scale in a model that included the type of group (studied or matched group), gender, age, body mass index, place of residence, education level, employment status, economic situation, and weight loss of the patients are presented in Table 5, Table 6, Table 7, Table 8, Table 9, Table 10, Table 11, Table 12, Table 13 and Table 14, showing the results within each scale and measure. The analysis showed that, depending on the scale from EORTC QLQ C30, different factors influenced the scores obtained, with patient weight loss, type of group (with or without enteral nutrition), and age being the most influential factors. Additional stepwise multiple regression analysis for Global Health Status (QoL) of EORTC QLQ C30 in a model with 5 functional scales (physical, role, cognitive, emotional, and social) and 3 symptom scales (fatigue, pain, and nausea/vomiting) is shown in Table 14. Additional analysis revealed that, depending on the Global Health Status (QoL) of EORTC QLQ C30, different factors influenced the obtained values, with the functional scales (emotional, social, and physical function) and symptom scales (vomiting/nausea) having the greatest influence.

## 4. Discussion

The present study shows that the EORTC QLQ C30 questionnaire maintained its psychometric properties in the studied population of patients with cancers of the head and neck and upper gastrointestinal tract. Considering the reference values for the physical scale, it should be noted that cancer site and the stage of the disease in general may influence the obtained scores. The scores for the EORTC QLQ C30 questionnaire could be generally higher for men (PF = 78.5) than for women (PF = 74.7), for patients < 50 years (PF = 80.2) than for those ≥70 years old (PF = 72.1), and for stage I-II (PF = 84.5) than stage III–IV (PF = 71.1) [17] but not always significantly different [25].

In the study conducted, type of group (studied or matched group) and age were the most influential factors, whereas, interestingly, gender was not reported as a statistically significant determinant. Gender has been reported as a determinant of quality of life in cancer patients in a number of studies [26,27], but there are also studies indicating a lack of association [28], while others indicate that this association cannot be generalized to all patients [29]. Also, in the Danish population, gender did not seem to be a clinically important determinant of the results obtained, as statistically significant differences were found in five out of fifteen scales within the EORTC QLQ C30 questionnaire [30]. Despite the fact that the results for some scales differed from the reference values, clinical significance must be taken into consideration.

Giesinger et al. [25] found no significant interaction between grouping variables (age, sex, tumour stage, treatment status, and country) and QLQ C30 scores in predicting cases defined by the criteria for the threshold for clinical significance. It should be noted that the mentioned study examined a mixed population, including patients from Poland. Moreover, the analysis of the influence of the country showed that patients from Poland differed significantly from those in other countries in terms of EF and FA [25]. The item-scale correlation matrix showed moderate to high correlation, the magnitude of which depended on the scale and ranged from 0.23 to 0.72. As presented in Table 4, the strongest relationships were found between the role function and fatigue scales, and the weakest between the pain and vomiting/nausea scales. It should be noted, however, that the EORTC organization found that some of the scales were correlated with each other, as expected, because they measured different dimensions of the construct “quality of life” [31]. Comparing these results with a similar study by Sherman et al. [32] conducted in a sample of patients with head and neck cancer yields comparable correlations.

The results of the stepwise multiple regression analysis for the appetite loss scale of EORTC QLQ C30 showed that patient weight loss, use of enteral nutrition, and age were the most important factors affecting the results. This finding is significant in light of the research findings of the study by McKerna et al. [33], in which the authors stated that appetite loss can be treated as a prognostic factor in cancer patients. Moreover, Blazeby et al. [34] reported in their study on patients with oesophageal cancer that appetite loss was associated with lower survival. However, it should be mentioned that this association is not generally true for cancer patients and that even an inverse association can be observed. In obese women with early breast cancer, weight loss was associated with more favourable changes in quality of life. Regardless of these conflicting results, the recent systematic review and meta-analysis by Hanna et al. [35] emphasized the role of skeletal mass loss, indicating that there is a general association between skeletal mass and quality of life.

Considering the negative influence of the loss of body mass [36] in cancer patients, one of the motives to use enteral nutrition, if only possible, is to prevent the loss of body mass or at least to delay the progression of body mass loss [14]. This could also explain the observed relationship between applied enteral nutrition and quality of life, as the re-cent systematic review by Gliwska et al. [37] indicated that enteral nutrition should be applied whenever possible to prevent and treat malnutrition in cancer patients but also to improve patients’ quality of life. Last but not least, it should be emphasized that the observation that enteral nutrition in palliative care is associated with a decreased quality of life [38] is not due to its negative influence but to the fact that it is used in patients with advanced cancer and in the worst clinical situation [39]. In view of this, enteral nutrition should be administered as soon as possible to prevent loss of body mass and maintain quality of life. The recent systematic review and meta-analysis by Chow et al. [40] con-firms that enteral nutrition should be administered when oral nutrition alone is inadequate and the patient is not meeting his or her energy needs.

The final influencing factor that has the greatest impact on quality of life, age, is also associated with loss of body mass [41]. Taking this into account, it can be considered as a complementary element of the observed association because loss of body mass is associated with age, but it can be reversed by enteral nutrition; so, all factors together are responsible for the body mass of cancer patients. In addition, aging is the other factor associated with cancer prognosis, with survival decreasing with age [42]. This is also reflected in the observed association between age and the need for enteral nutrition in cancer patients [43]. On the basis of the described association, the recommendation of enteral nutrition to patients must result not only from the increase in the patient’s chances but also from the need to improve their quality of life, which is especially necessary when patients do not want enteral nutrition, which is one of the common obstacles to the use of this type of treatment [44]. Noteworthy, clinical nutrition in oncology is often considered as a life-saving procedure, for example, in patients with cachexia or dysphagia [45]. Moreover, adequate nutritional status in many cases influences the ability to start or continue oncological treatment. Educating healthcare practitioners, patients, and the broader community about the impact of enteral nutrition on the prognosis, life expectancy, and quality of life in head and neck or upper gastrointestinal cancers appears to be of considerable importance.

Our study has several limitations, one of them being the sample size. Nevertheless, pair-matched analysis requires highly specific criteria for patient inclusion. It was deemed pertinent to assess the impact of nutritional status on the quality of life within the two study groups. However, a significant proportion of the participants were diagnosed with malnutrition, which impeded the feasibility of conducting a more comprehensive and in-depth analysis. Another limitation of this study was the absence of randomization which could influence the results.

## 5. Conclusions

This study revealed that the studied group more often received lower outcomes in functional scales while having higher scores in the symptomatic scales. Moreover, it revealed that the studied group more often experienced appetite loss, dyspnoea, or financial difficulties. For the patients with cancer of the head and neck and upper gastrointestinal tract, age, weight loss, and enteral nutrition are the most important factors affecting their quality of life. Therefore, weight loss should be counteracted with various therapeutic options, including mandatory enteral nutrition when needed, and such an approach is especially necessary in elderly patients.

## Figures and Tables

**Table 1:** The socio-demographic characteristics of patients stratified on studied group and matched group.

		Total	Studied Group	Matched Group	*p*
Gender	Male	26 (52%)	13 (52%)	13 (52%)	0.7773 *
	Female	24 (48%)	12 (48%)	12 (48%)	
Level of education	Higher education	15 (30%)	5 (20%)	10 (40%)	0.6504
	Secondary education	22 (44%)	12 (48%)	10 (40%)	
	Vocational education	13 (26%)	8 (32%)	5 (20%)	
Place of residence	Rural area	23 (46%)	11 (44%)	12 (48%)	0.4900
	Town with up to 20,000 inhabitants	2 (4%)	1 (4%)	1 (4%)	
	City with 20,000 to 100,000 inhabitants	9 (18%)	7 (28%)	2 (8%)	
	City with 100,000 to 500,000 inhabitants	2 (4%)	1 (4%)	1 (4%)	
	City with over 500,000 inhabitants	14 (28%)	5 (20%)	9 (36%)	
Employment status	Employed full-time	11 (22%)	4 (16%)	7 (28%)	0.7608
	Employed part-time	1 (2%)	1 (4%)	0 (0%)	
	Pension	3 (6%)	1 (4%)	2 (8%)	
	Retirement	29 (58%)	14 (56%)	15 (60%)	
	Unemployed	6 (12%)	5 (20%)	1 (4%)	
Economic status	Very good	11 (22%)	2 (8%)	9 (36%)	0.0323
	Good	15 (30%)	10 (40%)	5 (20%)	
	Neither good nor bad	13 (26%)	9 (36%)	4 (16%)	
	Bad	5 (10%)	4 (16%)	1 (4%)	
	Very bad	6 (12%)	0 (0%)	6 (24%)	

* Chi^2^ with Yates’ correction.

**Table 2:** Age of patients stratified on studied group and matched group.

	Mean (SD)	95% CI	Median (IQR)	*p*
Total	63.0 (11.8)	59.64–66.36	65.5 (16.0)	
Studied group	62.92 (12.02)	58.19–67.97	66.0 (16.0)	0.9690
Matched group	63.1 (11.83)	57.96–67.88	64.0 (14.0)	

**Table 3:** Descriptive statistics and scale reliability of the EORTC QLQ C30 compared to reference values for (1) all cancer patients and all stages of cancer [20] and (2) Polish general population [21].

Measures	Scale	Mean ± SD	Median (IQR)	95%CI	(1) All Cancer Patients	(2) General Population
Functional scales	Physical	67.33 ± 25.71	80 (40)	(60.03–74.64)	lower	lower
	Role	53.67 ± 40.17	66.67 (100)	(42.25–65.08)	lower	lower
	Cognitive	68.67 ± 29.48	83.33 (66.67)	(60.29–77.04)	lower	lower
	Emotional	56 ± 27.97	58.33 (41.67)	(48.05–63.95)	lower	lower
	Social	48 ± 29.48	50 (33.34)	(39.62–56.38)	lower	lower
Global health status		39.67 ± 18.79	41.67 (25)	(34.33–45.01)	lower	lower
Symptom Scales	Fatigue	44.89 ± 30.45	33.33 (33.34)	(36.24–53.54)	similar	higher
	Pain	41.67 ± 34.38	33.33 (50)	(31.9–51.44)	higher	higher
	Vomiting/nausea	12.33 ± 20.15	0 (16.67)	(6.61–18.06)	similar	higher
Single-item measures	Dyspnoea	22.67 ± 31.18	0 (33.33)	(13.8–31.53)	similar	higher
	Insomnia	48 ± 34.44	33.33 (33.34)	(38.21–57.79)	similar	higher
	Appetite loss	38.67 ± 36.49	33.33 (66.67)	(28.3–49.04)	higher	higher
	Constipation	22.67 ± 29.69	0 (33.33)	(14.23–31.11)	similar	higher
	Diarrhoea	15.33 ± 31.74	0 (0)	(6.31–24.35)	similar	similar
	Financial difficulties	29.33 ± 31.33	33.33 (33.33)	(20.43–38.24)	higher	higher

**Table 4:** EORTC QLQ C30: Item-scale correlation matrix.

	PF	RF	CF	EF	SF	F	P	VN	QoL
Physical functioning (PF)	-								
Role functioning (RF)	0.53	-							
Cognitive functioning (CF)	0.56	0.56	-						
Emotional functioning (EF)	0.37	0.49	0.70	-					
Social functioning (SF)	0.41	0.64	0.46	0.57	-				
Fatigue (F)	−0.47	−0.72	−0.63	−0.60	−0.61	-			
Pain (P)	−0.53	−0.48	−0.62	−0.49	−0.54	0.62	-		
Vomiting/nausea (VN)	−0.33	−0.40	−0.48	−0.32	−0.43	0.45	0.23	-	
Global health status (QoL)	0.50	0.44	0.49	0.38	0.56	−0.50	−0.37	−0.29	-

**Table 5:** Results of the stepwise multiple regression analysis for physical scale of EORTC QLQ C30 in a model including type of group (studied or matched group), gender, age, body mass index, place of residence, level of education, employment status, economic situation, and weight loss of patients as variables within the population of combined studied and matched groups (*n* = 50).

	Unstandardized Coefficients		Standardized Coefficients β	*p*
	β	SE		
Constant			1.0780	<0.0001
Weight loss of patients	0.4195	0.1284	0.0247	0.0020
Economic situation	0.2795	0.1284	0.1047	0.0345

SE—standard error.

**Table 6:** Results of the stepwise multiple regression analysis for emotional scale of EORTC QLQ C30 in a model including type of group (studied or matched group), gender, age, body mass index, place of residence, level of education, employment status, economic situation, and weight loss of patients as variables within the population of combined studied and matched groups (*n* = 50).

	Unstandardized Coefficients		Standardized Coefficients β	*p*
	β	SE		
Constant			2.4912	<0.0001
Age	−0.3227	0.1366	−0.0129	0.0223

SE—standard error.

**Table 7:** Results of the stepwise multiple regression analysis for vomiting/nausea scale of EORTC QLQ C30 in a model including type of group (studied or matched group), gender, age, body mass index, place of residence, level of education, employment status, economic situation, and weight loss of patients as variables within the population of combined studied and matched groups (*n* = 50).

	Unstandardized Coefficients		Standardized Coefficients β	*p*
	β	SE		
Constant			1.1600	<0.0001
Type of group (studied/matched)	0.3333	0.1361	0.3200	0.0180

SE—standard error.

**Table 8:** Results of the stepwise multiple regression analysis for dyspnoea scale of EORTC QLQ C30 in a model including type of group (studied or matched group), gender, age, body mass index, place of residence, level of education, employment status, economic situation, and weight loss of patients as variables within the population of combined studied and matched groups (*n* = 50).

	Unstandardized Coefficients		Standardized Coefficients β	*p*
	β	SE		
Constant			1.1600	<0.0001
Type of group (studied/matched)	0.2837	0.1384	0.2800	0.0459

SE—standard error.

**Table 9:** Results of the stepwise multiple regression analysis for insomnia scale of EORTC QLQ C30 in a model including type of group (studied or matched group), gender, age, body mass index, place of residence, level of education, employment status, economic situation, and weight loss of patients as variables within the population of combined studied and matched groups (*n* = 50).

	Unstandardized Coefficients		Standardized Coefficients β	*p*
	β	SE		
Constant			0.5286	0.0963
Age	0.3097	0.1372	0.0110	0.0286

SE—standard error.

**Table 10:** Results of the stepwise multiple regression analysis for appetite loss scale in of EORTC QLQ C30 in a model including type of group (studied or matched group), gender, age, body mass index, place of residence, level of education, employment status, economic situation, and weight loss of patients as variables within the population of combined studied and matched groups (*n* = 50).

	Unstandardized Coefficients		Standardized Coefficients β	*p*
	β	SE		
Constant			1.5804	<0.0001
Weight loss of patients	−0.2945	0.1379	−0.0176	0.0379

SE—standard error.

**Table 11:** Results of the stepwise multiple regression analysis for constipation scale of EORTC QLQ C30 in a model including type of group (studied or matched group), gender, age, body mass index, place of residence, level of education, employment status, economic situation, and weight loss of patients as variables within the population of combined studied and matched groups (*n* = 50).

	Unstandardized Coefficients		Standardized Coefficients β	*p*
	β	SE		
Constant			1.2930	<0.0001
Weight loss of patients	0.3450	0.1355	0.0213	0.0141

SE—standard error.

**Table 12:** Results of the stepwise multiple regression analysis for diarrhoea scale of EORTC QLQ C30 in a model including type of group (studied or matched group), gender, age, body mass index, place of residence, level of education, employment status, economic situation, and weight loss of patients as variables within the population of combined studied and matched groups (*n* = 50).

	Unstandardized Coefficients		Standardized Coefficients β	*p*
	β	SE		
Constant			1.4427	<0.0001
Education level	0.2833	0.1384	0.1619	0.0462

SE—standard error.

**Table 13:** Results of the stepwise multiple regression analysis for financial difficulties scale of EORTC QLQ C30 in a model including type of group (studied or matched group), gender, age, body mass index, place of residence, level of education, employment status, economic situation, and weight loss of patients as variables within the population of combined studied and matched groups (*n* = 50).

	Unstandardized Coefficients		Standardized Coefficients β	*p*
	β	SE		
Constant			1.7250	<0.0001
Economic situation	−0.3227	0.1366	−0.1250	0.0223

SE—standard error.

**Table 14:** Results of the stepwise multiple regression analysis for Global Health Status (QoL) of EORTC QLQ C30 in a model including type of group (studied or matched group), gender, age, body mass index, place of residence, level of education, employment status, economic situation, and weight loss of patients as variables within the population of combined studied and matched groups (*n* = 50).

	Unstandardized Coefficients		Standardized Coefficients β	*p*
	β	SE		
Constant			0.2306	0.4754
Functional scales—Emotional functioning	0.3192	0.1208	0.2224	0.0113
Functional scales—Social functioning	0.3490	0.1218	0.2835	0.0063
Functional scales—Physical functioning	0.3867	0.1220	0.2653	0.0027
Symptom scales—Vomiting/nausea	0.2928	0.1264	0.1983	0.0251

SE—standard error.

## Data Availability

The data presented in this study are available on request from the corresponding author. The data are not publicly available due to ethical restrictions.

## References

[1] Siegel R.L., Miller K.D., Wagle N.S., Jemal A. (2023). Cancer statistics. Cancer J. Clin..

[2] Soerjomataram I., Bray F. (2021). Planning for tomorrow: Global cancer incidence and the role of prevention 2020–2070. Nat. Rev. Clin. Oncol..

[3] Nayak M.G., George A., Vidyasagar M.S., Mathew S., Nayak S., Nayak B.S., Shashidhara Y.N., Kamath A. (2017). Quality of Life among Cancer Patients. Indian J. Palliat. Care.

[4] Kennifer S., Alexander S., Pollak K., Jeffreys A., Olsen M., Rodriguez K., Arnold R., Tulsky J. (2009). Negative emotions in cancer care: Do oncologists’ responses depend on severity and type of emotion?. Patient Educ. Couns..

[5] Shrestha A., Martin C., Burton M., Walters S., Collins K., Wyld L. (2019). Quality of life versus length of life considerations in cancer patients: A systematic literature review. Psychooncology.

[6] Salvetti M.G., Machado C.S.P., Donato S.C.T., Silva A.M.D. (2020). Prevalence of symptoms and quality of life of cancer patients. Braz. J. Nurs..

[7] Arends J., Baracos V., Bertz H., Bozzetti F., Calder P.C., Deutz N.E.P., Erickson N., Laviano A., Lisanti M.P., Lobo D.N. (2017). ESPEN expert group recommendations for action against cancer-related malnutrition. Clin. Nutr..

[8] Cederholm T., Jensen G.L., Correia M., Gonzalez M.C., Fukushima R., Higashiguchi T., Baptista G., Barazzoni R., Blaauw R., Coats A.J.S. (2019). GLIM criteria for the diagnosis of malnutrition—A consensus report from the global clinical nutrition community. J. Cachexia Sarcopenia Muscle.

[9] Schneider S.M., Correia M.I.T.D. (2020). Epidemiology of weight loss, malnutrition and sarcopenia: A transatlantic view. Nutrition.

[10] Beirer A. (2021). Malnutrition and cancer, diagnosis and treatment. Mag. Eur. Med. Oncol..

[11] Ortega M., Torres J. (2023). The Cleveland Clinic: Gastrointestinal Symptoms in Cancer Patients with Advanced Disease. The Cleveland Clinic: Improving the Patient Experience.

[12] Rangwala F., Zafar S.Y., Abernethy A.P. (2012). Gastrointestinal symptoms in cancer patients with advanced disease: New methodologies, insights, and a proposed approach. Curr. Opin. Support. Palliat. Care.

[13] Poulsen G.M., Pedersen L.L., Østerlind K., Bæksgaard L., Andersen J.R. (2014). Randomized trial of the effects of individual nutritional counseling in cancer patients. Clin. Nutr..

[14] Muscaritoli M., Arends J., Bachmann P., Baracos V., Barthelemy N., Bertz H., Bozzetti F., Hütterer E., Isenring E., Kaasa S. (2021). ESPEN practical guideline: Clinical Nutrition in cancer. Clin. Nutr..

[15] McCurdy B., Nejatinamini S., Debenham B.J., Álvarez-Camacho M., Kubrak C., Wismer W.V., Mazurak V.C. (2019). Meeting minimum ESPEN energy recommendations is not enough to maintain muscle mass in head and neck cancer patients. Nutrients.

[16] Prado C.M., Laviano A., Gillis C., Sung A.D., Gardner M., Yalcin S., Dixon S., Newman S.M., Bastasch M.D., Sauer A.C. (2022). Examining guidelines and new evidence in oncology nutrition: A position paper on gaps and opportunities in multimodal approaches to improve patient care. Support. Care Cancer.

[17] Wanden-Berghe C., Patino-Alonso M.-C., Galindo-Villardón P., Sanz-Valero J. (2019). Complications associated with enteral nutrition: CAFANE study. Nutrients.

[18] Thomas A., Sowerbutts A.M., Burden S.T. (2020). The impact of home enteral feeding on the daily lives of people with head and neck cancer: A metasynthesis of qualitative studies. J. Hum. Nutr. Diet..

[19] Tomaszewski K.A., Püsküllüoğlu M., Biesiada K., Bochenek J., Nieckula J., Krzemieniecki K. (2013). Validation of the polish version of the eortc QLQ-C30 and the QLQ-OG25 for the assessment of health-related quality of life in patients with esophagi-gastric cancer. J. Psychosoc. Oncol..

[20] Scott N., Fayers P., Aaronson N., Bottomley A., de Graeff A., Groenvold M., Gundy C., Koller M., Petersen M.A., Sprangers M.A. (2008). EORTC QLQ-C30. Ref Values.

[21] Nolte S., Liegl G., Petersen M., Aaronson N., Costantini A., Fayers P., Groenvold M., Holzner B., Johnson C., Kemmler G. (2019). General population normative data for the EORTC QLQ-C30 health-related quality of life questionnaire based on 15,386 persons across 13 European countries, Canada and the Unites States. Eur. J. Cancer.

[22] Grulke N., Albani C., Bailer H. (2012). Quality of life in patients before and after haematopoietic stem cell transplantation measured with the European Organization for Research and Treatment of Cancer (EORTC) Quality of Life Core Questionnaire QLQ-C30. Bone Marrow Transpl..

[23] Nunnally J.C., Bernstein I.H. (1994). Psychometric Theory.

[24] World Health Organization (1992). CIM-10. Classification Statistique Internationale des Maladies et des Problèmes de Santé Connexes: Dixième Révision.

[25] Giesinger J.M., Kuijpers W., Young T., Tomaszewski K.A., Friend E., Zabernigg A., Holzner B., Aaronson N.K. (2016). Thresholds for clinical importance for four key domains of the EORTC QLQ-C30: Physical functioning, emotional functioning, fatigue and pain. Health Qual. Life Outcomes.

[26] Laghousi D., Jafari E., Nikbakht H., Nasiri B., Shamshirgaran M., Aminisani N. (2019). Gender differences in health-related quality of life among patients with colorectal cancer. J. Gastrointest. Oncol..

[27] Pud D. (2011). Gender differences in predicting quality of life in cancer patients with pain. Eur. J. Oncol. Nurs..

[28] Robson P.C., Dietrich M.S., Akard T.F. (2021). Associations of Age, Gender, and Family Income with Quality of Life in Children With Advanced Cancer. J. Pediatr. Oncol. Nurs..

[29] Koch M., Hjermstad M.J., Tomaszewski K., Tomaszewska I., Hornslien K., Harle A., Arraras J., Morag O., Pompili C., Ioannidis G. (2020). Gender effects on quality of life and symptom burden in patients with lung cancer: Results from a prospective, cross-cultural, multi-center study. J. Thorac. Dis..

[30] Juul T., Petersen M.A., Holzner B., Laurberg S., Christensen P., Grønvold M. (2014). Danish population-based reference data for the EORTC QLQ-C30: Associations with gender, age and morbidity. Qual. Life Res..

[31] Aaronson N.K., Bullinger M., Ahmedzai S. (1988). A modular approach to quality-of-life assessment in cancer clinical trials. Cancer Clinical Trials: A Critical Appraisal.

[32] Sherman A.C., Simonton S., Adams D.C., Vural E., Owens B., Hanna E. (2000). Assessing quality of life in patients with head and neck cancer: Cross-validation of the European Organization for Research and Treatment of Cancer (EORTC) Quality of Life Head and Neck module (QLQ-H&N_35_). Arch. Otolaryngol. Neck Surg..

[33] McKernan M., McMillan D.C., Anderson J.R., Angerson W.J., Stuart R.C. (2008). The relationship between quality of life (EORTC QLQ-C30) and survival in patients with gastro-oesophageal cancer. Br. J. Cancer.

[34] Blazeby J.M., Williams M.H., Brookes S.T., Alderson D., Farndon J.R. (1995). Quality of life measurement in patients with oesophageal cancer. Gut.

[35] Hanna L., Nguo K., Furness K., Porter J., Huggins C.E. (2022). Association between skeletal muscle mass and quality of life in adults with cancer: A systematic review and meta-analysis. J. Cachexia Sarcopenia Muscle.

[36] Di Meglio A., Michiels S., Jones L.W., El-Mouhebb M., Ferreira A.R., Martin E., Matias M., Lohmann A.E., Joly F., Vanlemmens L. (2020). Changes in weight, physical and psychosocial patient-reported outcomes among obese women receiving treatment for early-stage breast cancer: A nationwide clinical study. Breast.

[37] Gliwska E., Guzek D., Przekop Z., Sobocki J., Głąbska D. (2021). Quality of Life of Cancer Patients Receiving Enteral Nutrition: A Systematic Review of Randomized Controlled Trials. Nutrients.

[38] Caro M.M.M., Laviano A., Pichard C. (2007). Impact of nutrition on quality of life during cancer. Curr. Opin. Clin. Nutr. Metab. Care.

[39] Prevost V., Grach M.C. (2012). Nutritional support and quality of life in cancer patients undergoing palliative care. Eur. J. Cancer Care.

[40] Chow R., Bruera E., Arends J., Walsh D., Strasser F., Isenring E., Del Fabbro E.G., Molassiotis A., Krishnan M., Chiu L. (2020). Enteral and parenteral nutrition in cancer patients, a comparison of complication rates: An updated systematic review and (cumulative) meta-analysis. Support Care Cancer.

[41] Volpi E., Nazemi R., Fujita S. (2004). Muscle tissue changes with aging. Curr. Opin. Clin. Nutr. Metab. Care.

[42] Lu C.-H., Lee S.-H., Liu K.-H., Hung Y.-S., Wang C.-H., Lin Y.-C., Yeh T.-S., Chou W.-C. (2018). Older age impacts on survival outcome in patients receiving curative surgery for solid cancer. Asian J. Surg..

[43] Sachdev S., Refaat T., Bacchus I.D., Sathiaseelan V., Mittal B.B. (2015). Age most significant predictor of requiring enteral feeding in head-and-neck cancer patients. Radiat. Oncol..

[44] Nasrah R., Van Der Borch C., Kanbalian M., Jagoe R.T. (2020). Defining barriers to implementation of nutritional advice in patients with cachexia. J. Cachexia Sarcopenia Muscle.

[45] Amano K., Maeda I., Ishiki H., Miura T., Hatano Y., Tsukuura H., Taniyama T., Matsumoto Y., Matsuda Y., Kohara H. (2021). Effects of enteral nutrition and parenteral nutrition on survival in patients with advanced cancer cachexia: Analysis of a multicenter prospective cohort study. Clin. Nutr..

